# Mesenteroaxial gastric volvulus in an elderly patient 5 years after Nissen fundoplication and hiatal hernia repair

**DOI:** 10.1093/jscr/rjae678

**Published:** 2024-11-06

**Authors:** Harpreet Gill, Angel Guan, Alexandra Nguyen, So Un Kim, Gunjan Bhat, Aldin Malkoc, Sunal Patel

**Affiliations:** Department of Surgery, Arrowhead Regional Medical Center, 400 N Pepper Ave, Colton, CA 92324, United States; Department of Surgery, Arrowhead Regional Medical Center, 400 N Pepper Ave, Colton, CA 92324, United States; Department of Surgery, Arrowhead Regional Medical Center, 400 N Pepper Ave, Colton, CA 92324, United States; Department of Surgery, Arrowhead Regional Medical Center, 400 N Pepper Ave, Colton, CA 92324, United States; Department of Surgery, Arrowhead Regional Medical Center, 400 N Pepper Ave, Colton, CA 92324, United States; Department of Surgery, Arrowhead Regional Medical Center, 400 N Pepper Ave, Colton, CA 92324, United States; Department of Surgery, Kaiser Permanente Medical Center, 2295 S Vineyard Avenue, Ontario, CA 91761, United States

**Keywords:** gastric volvulus, hernia, Nissen fundoplication

## Abstract

Gastric volvulus is a rare and potentially life-threatening condition that usually presents acutely and requires immediate intervention via either endoscopic or surgical detorsion. Most often, it presents secondary to a hiatal hernia, with herniation and torsion of the stomach through the hiatus. Only a small subset of patients present with gastric volvulus after Nissen fundoplication for hiatal hernia repair. We report the case of an elderly patient with a previous hiatal hernia repair with Nissen fundoplication who presented with an intra-abdominal gastric volvulus that developed over the course of several months. The combination of her surgical history and volvulus etiology made her disease rare. She was treated with laparoscopic surgery and gastropexy with gastrostomy tube placement, which led to the resolution of the upper gastrointestinal symptoms.

## Introduction

Gastric volvulus is a rare yet potentially life-threatening condition characterized by abnormal torsion of the stomach within the abdominal cavity, typically exceeding 180°. This torsion causes closed-loop obstruction, which can result in incarceration and strangulation. Classically, gastric volvulus is categorized based on axis of rotation [1]. Organoaxial volvulus, the most common type, is often associated with paraesophageal hernias and diaphragmatic eventration [2]. It involves rotation around the axis joining the gastroesophageal junction and pylorus, resulting in an ‘inverted’ stomach, with the greater curvature of the stomach positioned superiorly. Mesenteroaxial volvulus entails rotation of the pyloric area along the short axis, causing the posterior surface of the stomach to shift anteriorly. This is more often the result of ligament laxity [3].

Gastric volvulus may also be classified according to etiology. Primary gastric volvulus occurs when gastric ligament laxity arises from congenital or acquired causes [4]. Secondary gastric volvulus occurs in association with disorders of gastric anatomy or function, or abnormalities of adjacent organs such as the diaphragm or spleen. Hiatal hernias and diaphragmatic defects are commonly implicated in the pathogenesis of gastric volvulus, and only a small subset of volvulus cases occur in the absence of these predisposing factors [2, 5]. A few reported cases of gastric volvulus post-Nissen fundoplication have implicated intrathoracic stomach [6], postoperative adhesions [7], or postoperative gastric ligament laxity [8]. Here we present the rare case of an elderly patient with a history of hiatal hernia repair with Nissen fundoplication 5 years prior, who presented with mesenteroaxial gastric volvulus.

## Case report

**Figure 1:**
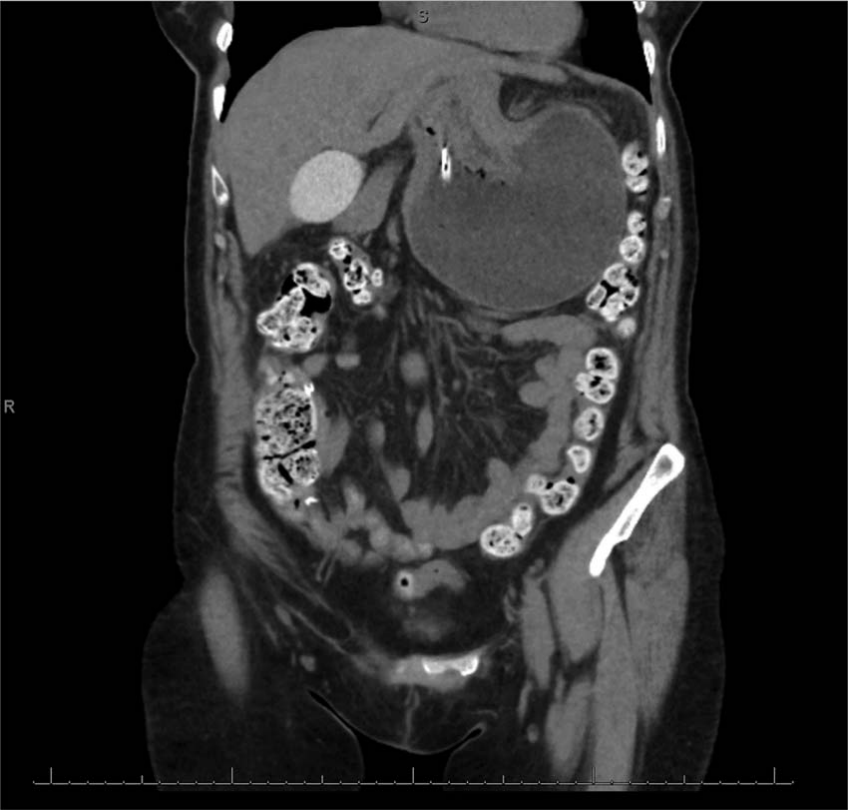
Coronal view of CT scan of abdomen and pelvis demonstrating gastric volvulus.

**Figure 2:**
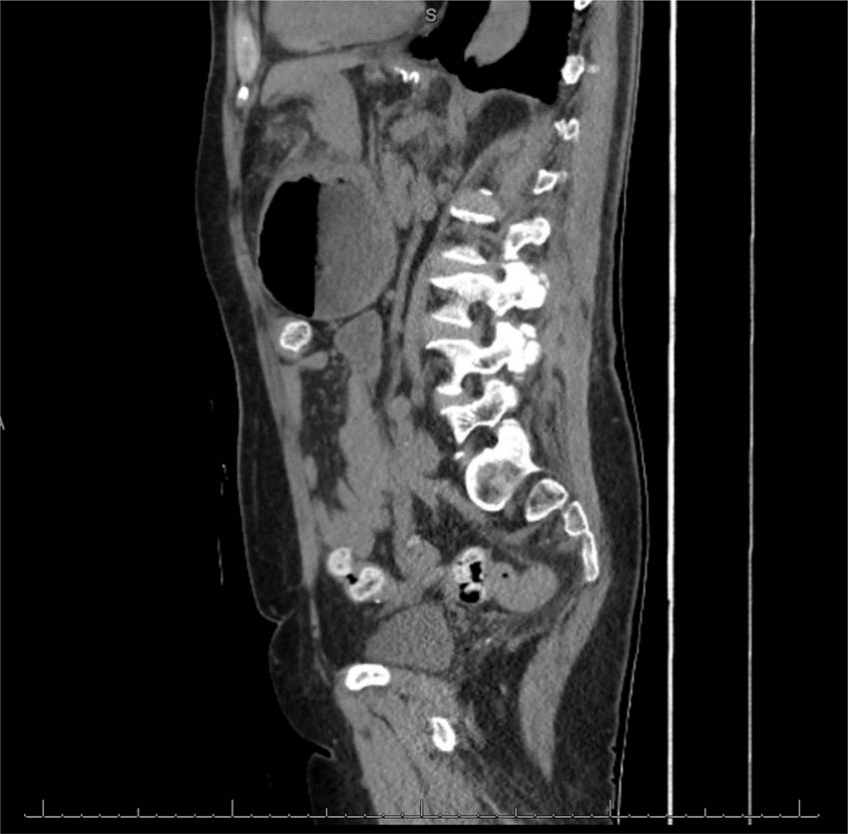
Sagittal view of CT scan of abdomen and pelvis demonstrating gastric volvulus.

**Figure 3:**
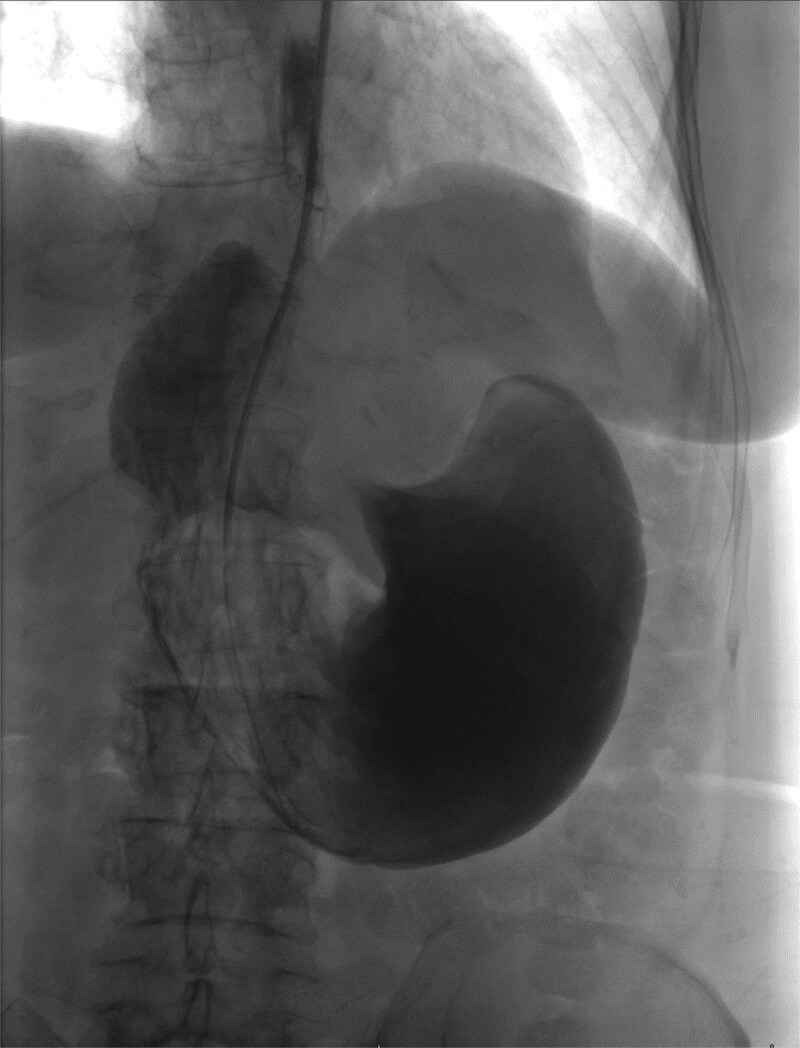
Upper GI study demonstrating no passage of contrast beyond stomach and 90° rotation.

The patient was a 74-year-old female with a history of hypertension, chronic migraines, hiatal hernia repair with Nissen fundoplication repair, and cecal volvulus treated with right hemicolectomy who presented with abdominal pain, nausea, and vomiting. Six months prior, she exhibited symptoms of bloating and nausea, which were treated with oral Reglan with some improvement. She reported vomiting episodes for 2 months that acutely worsened over the 4 days prior to presentation. Examination revealed a soft and mildly distended abdomen with tenderness in the epigastrium and left upper quadrant. Initial vital signs showed a temperature of 36.1°C, blood pressure of 149/88 mmHg, heart rate of 115 beats/min, and oxygen saturation of 95% on room air; tachycardia resolved with resuscitation. Labs were notable for a white blood cell count of 8.8 × 1000/mcL and hemoglobin of 14.2 g/dL. Sodium was 137 mEq/L, potassium was 3.3 mEq/L, and creatinine was 0.75 mg/dL. Computed tomography (CT) of the abdomen and pelvis (Figs 1 and 2) showed gastric volvulus without pneumatosis, significant wall thickening, or distention. Nasogastric tube was placed. An upper gastrointestinal fluoroscopy scan showed no passage of contrast beyond the stomach and demonstrated 90° rotation of the stomach (see Fig. 3). The patient underwent surgery that same day.

**Figure 4:**
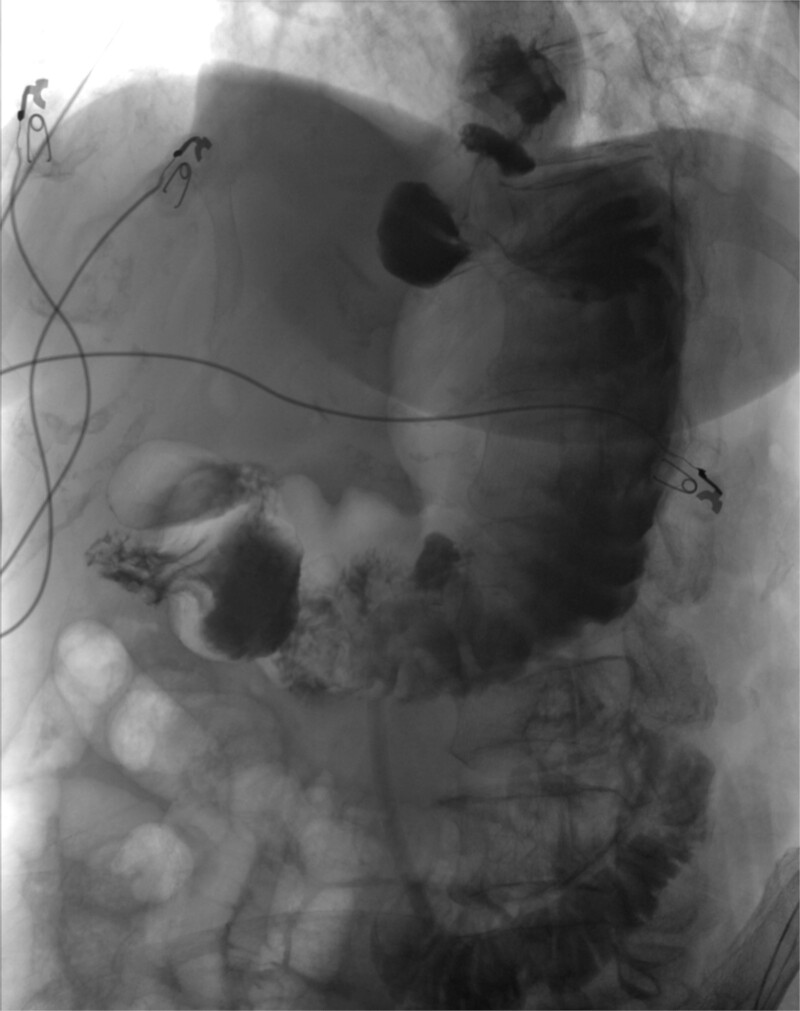
Upper GI study showing no leak after surgery.

Operative findings revealed gastric body herniation under the antrum and pylorus, causing 180° twisting of the stomach and obstruction of the antrum, consistent with mesenteroaxial volvulus. The gastrohepatic ligament was absent, and the greater curvature was nearly fully mobilized from the prior Nissen. The volvulus was laparoscopically reduced to restore normal anatomy, and a gastrostomy tube was placed to pexy the stomach to the abdominal wall to prevent recurrence. Finally, as an additional precaution, 10 units of Botox were injected into the pylorus to treat her possible gastroparesis diagnosed prior to her acute presentation. Postoperative course was uneventful. An upper GI study completed on postoperative Day 1 was negative for contrast leak (see Fig. 4). She was discharged home on postoperative Day 5. At her follow-up visit, she reported resolution of all symptoms.

## Discussion

Gastric volvulus is a rare but potentially life-threatening condition that should be promptly treated. A high index of suspicion is required for timely diagnosis as there is a wide spectrum of possible symptoms on presentation, especially in the setting of a history of other gastrointestinal complaints. Volvulus may present with Borchadt’s triad, which is significant epigastric pain, severe retching with inability to vomit, and inability to insert a nasogastric tube, or it may present with milder symptoms such as vague abdominal pain and nausea. Our patient didn’t meet the full criteria for Borchadt’s triad; though she had epigastric pain, she was able to vomit and a nasogastric tube was placed.

During surgery she was found to have mesenteroaxial volvulus without gastric ischemia. The absence of ischemia, in combination with her relatively non-toxic presentation, suggests that her volvulus progression was more chronic. Our hypothesis was that her lack of a gastrohepatic ligament and near-complete mobilization of the stomach’s greater curvature caused sufficient laxity to enable slow development of a volvulus. This is supported by the fact that prior to admission, she had been undergoing work-up with a gastroenterologist for abdominal pain and progressive dysphagia. She had been started on Reglan for suspected gastroparesis. Endoscopy in June 2023 demonstrated a large amount of semi-digested food within the stomach, without ulcers or strictures. An upper GI study in August 2023 was remarkable for mild distention of the stomach with contrast and debris, as well as delayed gastric emptying into the duodenal loop. Rather than gastroparesis, her symptoms were likely due to developing gastric volvulus.

Seventy percent of gastric volvulus occurs in conjunction with paraesophageal hernias. In this case, our patient actually underwent Nissen fundoplication in 2019. This is one of the few cases reported in the literature in which gastric volvulus occurred after Nissen fundoplication, and fundoplication was found to be intact at the time of surgery without hernia recurrence. This suggests that this patient had primary gastric volvulus, as no secondary causes were identified. This is consistent with our theory that post-Nissen laxity was the causative factor for her pathology.

## Conclusion

Rare cases of gastric volvulus can present after Nissen fundoplication without an obvious associated pathology. A high-level suspicion is necessary for timely diagnosis and treatment, especially in patients with nonspecific symptoms. In this case, we believe that laxity of the stomach after dissection during Nissen fundoplication enabled the development of a chronic mesenteroaxial gastric volvulus that was successfully treated with laparoscopic reduction and gastropexy.

## Data Availability

The authors declare that data supporting the findings of this study are available in this article.
